# Carbon Nanotube Enhanced Filtration and Dewatering of Kerosene

**DOI:** 10.3390/membranes12060621

**Published:** 2022-06-15

**Authors:** Sumona Paul, Mitun Chandra Bhoumick, Sagar Roy, Somenath Mitra

**Affiliations:** Department of Chemistry and Environmental Science, New Jersey Institute of Technology, Newark, NJ 07102, USA; sp2652@njit.edu (S.P.); mb777@njit.edu (M.C.B.); sagar.roy@njit.edu (S.R.)

**Keywords:** dewatering, filtration, hydrophobic, carbon nanotubes, fuel-water system

## Abstract

Current approaches to dewatering aviation fuel such as kerosene are adsorption by activated charcoal, gravity separation, etc. The objective of this work is to develop and demonstrate the filtration and dewatering of kerosene using a carbon nanotube immobilised membrane (CNIM). Highly hydrophobic membranes were prepared by immobilising carbon nanotube (CNTs) over polytetrafluoroethylene (PTFE) and polyvinylidene difluoride (PVDF) microfiltration membrane for the dewatering of ppm level water from kerosene. The effects of different CNT concentrations on membrane morphology, hydrophobicity, porosity, and permeability were characterised. After immobilising CNT into membranes, the contact angle increased by 9%, 16%, and 43% compared to unmodified 0.1 μm PTFE, 0.22 μm PTFE and 0.22 μm PVDF membranes, respectively. The CNIM showed remarkable separation efficiency for the fuel-water system. The micro/nano water droplets coalesced on the CNT surface to form larger diameters of water droplets detached from the membrane surface, leading to enhanced water rejection. In general, the water rejection increased with the amount of CNT immobilised while the effective surface porosity over pore length and flux decreased. PTFE base membrane showed better performance compared to the PVDF substrate. The CNIMs were fabricated with 0.1 and 0.22 μm PTFE at an optimised CNT loading of 3 and 6 wt.%, and the water rejection was 99.97% and 97.27%, respectively, while the kerosene fluxes were 43.22 kg/m^2^·h and 55.44 kg/m^2^·h respectively.

## 1. Introduction

Kerosene is an important fuel that is widely used in the aviation industry. Even a small amount of water contamination in kerosene can lead to an engine malfunction, which can be quite dangerous. Therefore, even trace amounts of water need to be removed from kerosene, which offers many challenges. The water in kerosene can exist in different forms, such as an immiscible layer or an emulsion. In a typical immiscible fuel-water mixture, as many as four different phases, namely the fuel, fuel-in-water, water-in-fuel, and pure water co-exist, and their separation can be challenging [1,2].

The efficient and economic separation of low concentration water from kerosene is important. Conventional techniques include gravity separation and skimming techniques that are easy to operate but cannot separate emulsions [1,2]. Other techniques such as different filters, chemical dosing, air floatation, chemical coagulation, electro-coagulation, electro-flotation, and biological processes have been used to separate oil-water systems [3,4,5,6]. One of the most common techniques is adsorption on activated carbon. However, the carbon materials are expensive and require complex regeneration [3,4,7].

Membrane-based technologies are becoming attractive alternatives for oil-water separation due to their low energy requirements, cost, custom fabrication, and wide applicability in a wide range of water contamination [4,5,8]. Conventional hydrophilic membranes have been used for gravity-driven separation but are not effective in restricting the permeation of free oil-water mixture or water in oil emulsion. Typically, hydrophobic and oleophilic (oil contact angle < 90°) are used in a crossflow filtration system to avoid any form of the barrier layer that prevents permeation of the organic phase [5]. However, these membranes are prone to fouling by oil during demulsification.

There have been significant efforts in developing nanostructured membranes in various applications, and of particular interest have been carbon nanotubes (CNTs) [7,9,10,11,12]. Recently, we have demonstrated that immobilising CNTs in different types of membranes (referred to as CNIM) alters the solute-membrane interactions [13]. These have been used in diverse processes such as desalination, solvent recovery, and concentrating trace amount contaminants [14,15,16]. The CNIM-based membranes have also been used for generating medical-grade water by rejecting endotoxin [17]. We have also shown that hydrophobic CNIM can be used in filtration, where the CNTs serve as the nucleus for water agglomeration [13]. Therefore, the immobilisation of CNTs can be used in different applications for selectively removing water, especially from fuels, which is an important application. The objective of this project was to study the dewatering of kerosene at ppm level using carbon nanotube immobilised hydrophobic membranes.

## 2. Experimental

### 2.1. Materials and Methods

Kerosene (Thermo Fisher Scientific, Ward Hill, MA, USA), Deionised water (Barnstead 5023, Dubuque, IA, USA), and MWCNTs (Cheap Tubes Inc., Brattleboro, VT, USA) were used in this study. The average diameter and length of the CNTs were ~40 nm and 15 nm, respectively. Porous composite polytetrafluoroethylene (PTFE) membranes on a polypropylene support layer with two different pore sizes (ANOW, 0.22 μm pore size, 74% porosity and 0.1 μm pore size, 68% porosity) and polyvinylidene difluoride (PVDF) membrane (Membrane Solutions, 0.22 μm pore size, 74% porosity) were used in this study.

### 2.2. CNIM Fabrication Process

Due to the agglomeration tendency of the CNTs, uniform dispersion in a solvent was an important consideration for CNIM fabrication [18,19]. CNTs (1.5−15 mg) were dispersed in a solution containing 15 mL acetone along with a small amount (0.15~0.2 mg) of polyvinylidene difluoride (PVDF) and sonicated for three hours. The PVDF solution acted as a binder during nanomaterials’ immobilisation. The sonicated PVDF-CNTs dispersion was then coated over the PTFE and PVDF membranes by vacuum forced deposition. Here a low vacuum was applied on the opposite side of the membrane to ensure uniform coating [13]. After that, membranes were allowed to dry overnight in a hood for the acetone to evaporate. Different amounts of CNTs were used to fabricate the CNIM, and an optimised CNT concentration was determined based on the different concentrations of the CNTs on the membrane surface. The CNIMs are referred to as CNIM-X, where X is the weight percentage of CNTs in the membrane.

### 2.3. Filtration Procedure

**Figure 1:**
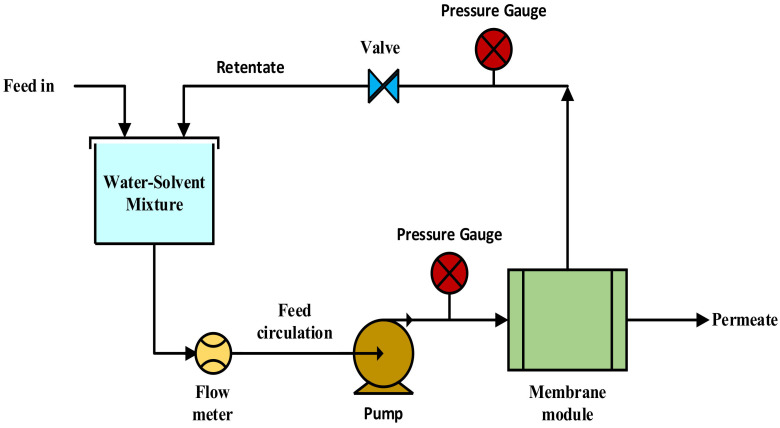
Schematic of dewatering kerosene by membrane filtration.

The concentration of water used in the kerosene feed mixture was in the range of 50–500 ppm. Different feed concentrations were prepared using a magnetic stirrer at 250–300 rpm for 10 min and 150–200 rpm stirring was maintained throughout the experiment. To reduce heat loss, this membrane cell was constructed using thick PTFE. The membrane module has two sides (Feed and Permeate), each with a 8.5 mm inlet and output. The surface area of this circular PTFE membrane module is 11.6 cm^2^. The exposed module surface toward membrane sides is baffled spaced to ensure proper fluid distribution. A gear pump (Cole Parmer, Vernon Hills, IL, USA) was used to pump the kerosene-water feed through the membrane module the retentate was collected in a tank while the feed was recycled. The pressure of the kerosene-water feed was controlled using a pressure control valve and measured by a pressure gauge. The feed pressure was varied between 6 to 15 psig at a constant feed flowrate of 40 mL/min monitored by a flow meter (Cole Parmer). The amount of water in permeates was measured using Gas Chromatography-Mass Spectroscopy (GC-MS) [20]. An HP 6890 GC coupled to an Agilent 5973N mass selective detector was used for GC–MS analysis. A schematic representation of the experimental setup is shown in Figure 1.

Solvent permeate flux (J_s_) and water rejection, R (%) [11,21] were defined and measured as:

(1) $$\mathrm{Flux}=\frac{\mathrm{Amount}\;\mathrm{permeate}\;\mathrm{through}\;\mathrm{the}\;\mathrm{membrane}\;\left(\mathrm{kg}\right)}{\mathrm{collection}\;\mathrm{time}\;\left(h\right)\times \;\mathrm{membrane}\;\mathrm{area}\;\left(m^{2}\right)}$$

(2) $$\mathrm{Rejection}\;\left(\%\right)=\frac{\mathrm{Water}\;\mathrm{concentration}\;\mathrm{in}\;\mathrm{feed}-\mathrm{Water}\;\mathrm{concentration}\;\mathrm{in}\;\mathrm{permeate}}{\mathrm{Water}\;\mathrm{concentration}\;\mathrm{in}\;\mathrm{feed}}\times 100\;$$

## 3. Results and Discussion

### 3.1. Scanning Electron Microscope of the Membranes

**Figure 2:**
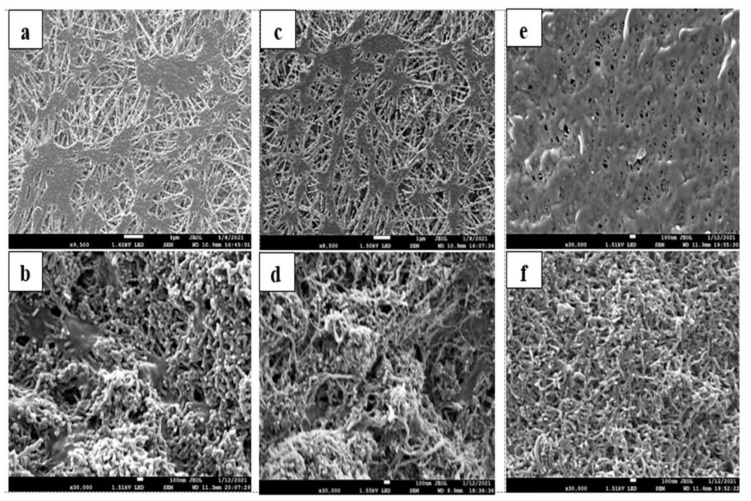
SEM images of PTFE and PVDF unmodified membranes and CNIM membranes: (**a**,**b**) unmodified PTFE (0.1 μm) and CNIM-3; (**c**,**d**) unmodified PTFE (0.22 μm) and CNIM-6; (**e**,**f**) unmodified PVDF (0.22 μm) and CNIM-6.

The CNIMs were studied using scanning electron microscopy (SEM, model JSM-7900F, JEOL USA Inc., Peabody, MA, USA). The membrane samples were cut into 0.5 cm long pieces and carbon-coated for SEM imaging. The SEM images of the original PTFE and PVDF base membranes and the CNIMs are presented in Figure 2a–f. The uniform distribution of CNTs was observed over the entire membrane surface. SEM images show the porous structure of the pristine membranes, and the presence of CNT coating on the membranes create channels across the membrane surface, facilitating solvent capillary flow, and repelling water cluster with their increased hydrophobicity.

### 3.2. Contact Angle Measurements

The water contact angles of the unmodified membranes and CNIMs are shown in Table 1. A Hamilton micro-syringe (0–10 µL) was used to drop water droplets (4 µL) onto the membrane surface, and a stage-mounted video camera was utilised to record the picture of the droplet. The average contact angle was reported for five different readings taken for each membrane. The presence of CNTs dramatically altered the contact angle [13,22,23]. The water contact angle for CNIMs was higher than that in the unmodified membranes, demonstrating the enhanced water repulsion in the CNIMs.

It is evident from Table 1 that the water contact angle, hence hreferences are ydrophobicity of the CNIMs, increases significantly during CNT immobilisation, and the water rejections reported here are higher than the unmodified membranes.

### 3.3. Membrane Porosity

The effective surface porosity over the effective pore length ($\frac{\in }{L_{p}})$ was measured by a gas permeation test [13,24]. The porosities of PTFE, PVDF membranes and CNIMs were measured using the gravimetric method, and the gas flux through the membrane was measured using a bubble flow meter at various pressures. For triplicate repeats, data points were gathered at various pressure levels, and average values were used to compute porosity. From the gas permeation test, the effective surface porosity over pore length (m^−1^) of PTFE 0.1 μm and CNIM-3 were found at 1.98 × 10^7^ and 2.93 × 10^5,^ respectively, while PTFE 0.22 μm and CNIM-6 were at 5.65 × 10^7^ and 1.61 × 10^7^. For the PVDF 0.22 μm, it was 1.41 × 10^6,^, and CNIM-6 showed a value of 1.00 × 10^6^. So, it is evident from the results that the effective porosity over pore length was lowered after the immobilising of CNTs. It was expected to lower the solvent permeation rate.

### 3.4. Thermal Gravimetric Analysis (TGA)

**Figure 3:**
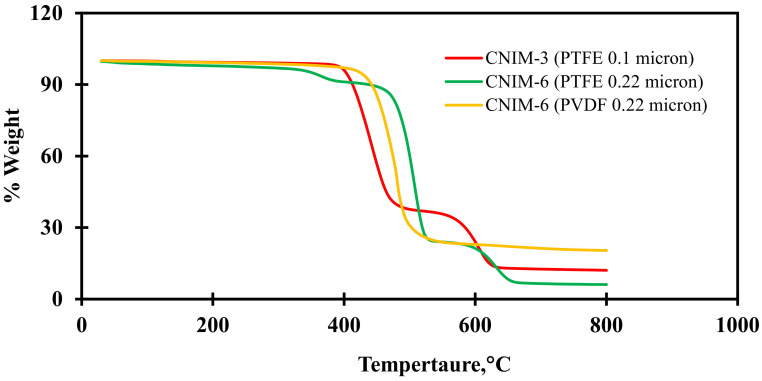
TGA curves for the CNIMs.

The thermal stability of CNIMs was investigated using the Perkin Elmer Pyris 7 TGA instrument at an isothermal heating rate of 10 °C/min in air. The membranes were found to be stable under working circumstances. The TGA results are presented in Figure 3. The weight loss at around 230 °C to 330 °C was due to the decomposition of PP as the supporting layer, while PTFE began to decompose at around 460–470 °C. Meanwhile, for the PVDF membrane, the degradation was seen only in a single time interval between 400–600 °C. This is because PVDF does not have any supporting layers. It was observed that the presence of CNTs provided thermal stability to the modified membrane. The TGA curve also shows the presence of CNTs on the membrane surface as % weight considering the base membrane.

### 3.5. Optimisation of CNTs Concentrations

The effect of CNT loading was studied in terms of flux and water rejection. Three concentrations of CNT loadings were studied. The effect of CNTs concentrations on kerosene flux and water rejection at 500 ppm water concentration (in feed) and at 10 psig transmembrane pressure is presented in Table 2.

From Table 2, it is clear that the CNTs concentrations had an important role in increasing the water contact angle and separation performance. With increasing the CNT concentration, the water rejection increased dramatically. However, after reaching an optimised concentration, the kerosene flux started reducing while the water rejection remained near the peak. This was possible due to the partial blockage of the membrane pores by the CNTs. For PTFE 0.1 μm, CNIM-3 was selected as the optimum dose of CNT where the water separation and the kerosene flux were considerable. Likewise, CNIM-6 was considered the optimum for 0.22 μm PTFE and PVDF membranes.

### 3.6. Effect of Transmembrane Pressure

**Figure 4:**
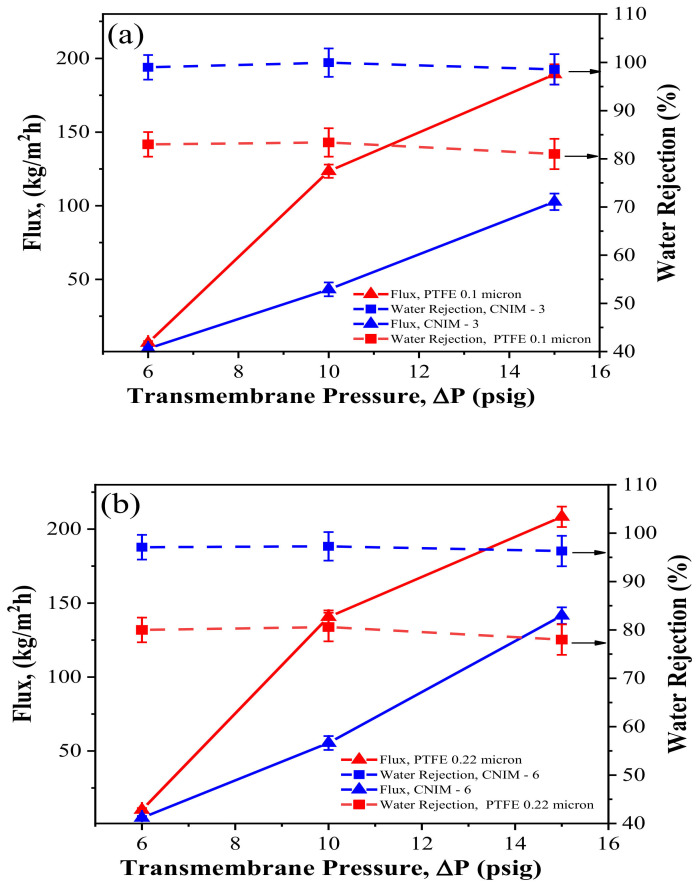

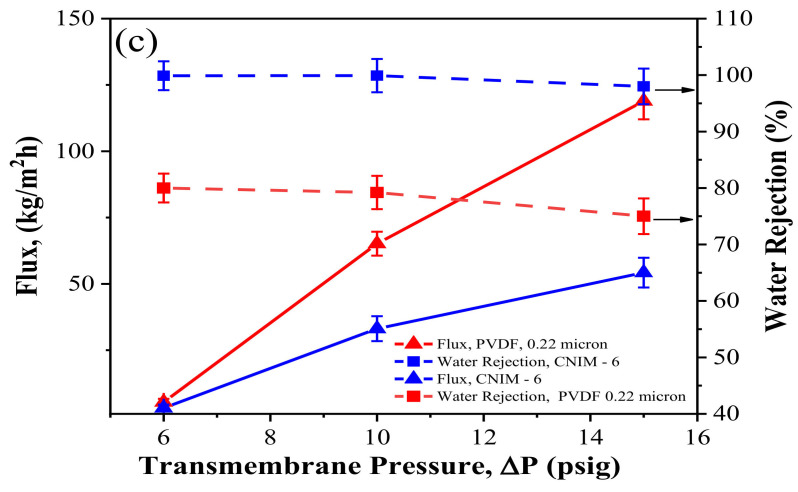
Effect of transmembrane pressure on kerosene flux and water rejection at a feed water concentration of 500 ppm and feed flowrate of 40 mL/min for (**a**) PTFE (0.1 μm) and CNIM-3; (**b**) PTFE (0.22 μm) and CNIM-6; and (**c**) PVDF (0.22 μm) and CNIM-6.

Figure 4 shows the effect of transmembrane pressure on solvent flux and water rejection of the CNIMs compared to the unmodified membranes. In all cases, the kerosene flux increased with increasing the transmembrane pressure.

As can be seen, the CNIMs exhibited comparable flux with significantly higher water rejection concerning the PTFE and PVDF membranes. The solvent flux is expected to improve with operating pressure. The CNIM showed better water separation efficiency than unmodified PTFE and PVDF membranes at all ∆P. At 10 psig, the water separation efficiency for the 0.1 µm PTFE membrane was 83% (Figure 4a), whereas 99.97% separation efficiency was observed for CNIM-3. Likewise, 0.22 µm PTFE exhibited 80.60% water rejection while CNIM-6 showed 97.27%, which was almost 21% higher than the unmodified PTFE at 10 psig. Also, the water rejection for the 0.22 µm PVDF membrane was lower (around 79%); however, the CNIM-6 showed 99.9% separation efficiency at a pressure of 10 psig. The preferential capillary force of CNTs for kerosene and inherent hydrophobicity of CNTs ensured higher water rejection [11,19,20,21,22,23]. However, when the applied pressure was very high, it exceeded the capillary pressure, and water could pass through the membranes [12,25,26,27,28]. As a result, at higher ∆P, the water flux increased faster than kerosene flux resulting in a decrease in water rejection. This is presented in Figure 4a–c. In Figure 4b, CNIM-6 prepared from PTFE 0.22 μm exhibited 97.27% water separation, whereas the kerosene flux was 55.44 kg/m^2^·h at 10 psig pressure. On the other hand, the PVDF membrane with the same pore size and CNIM-6 had a lower flux than the PTFE 0.22 μm and its CNIM-6, shown in Figure 4c.

### 3.7. Effect of Water Concentration

**Figure 5:**
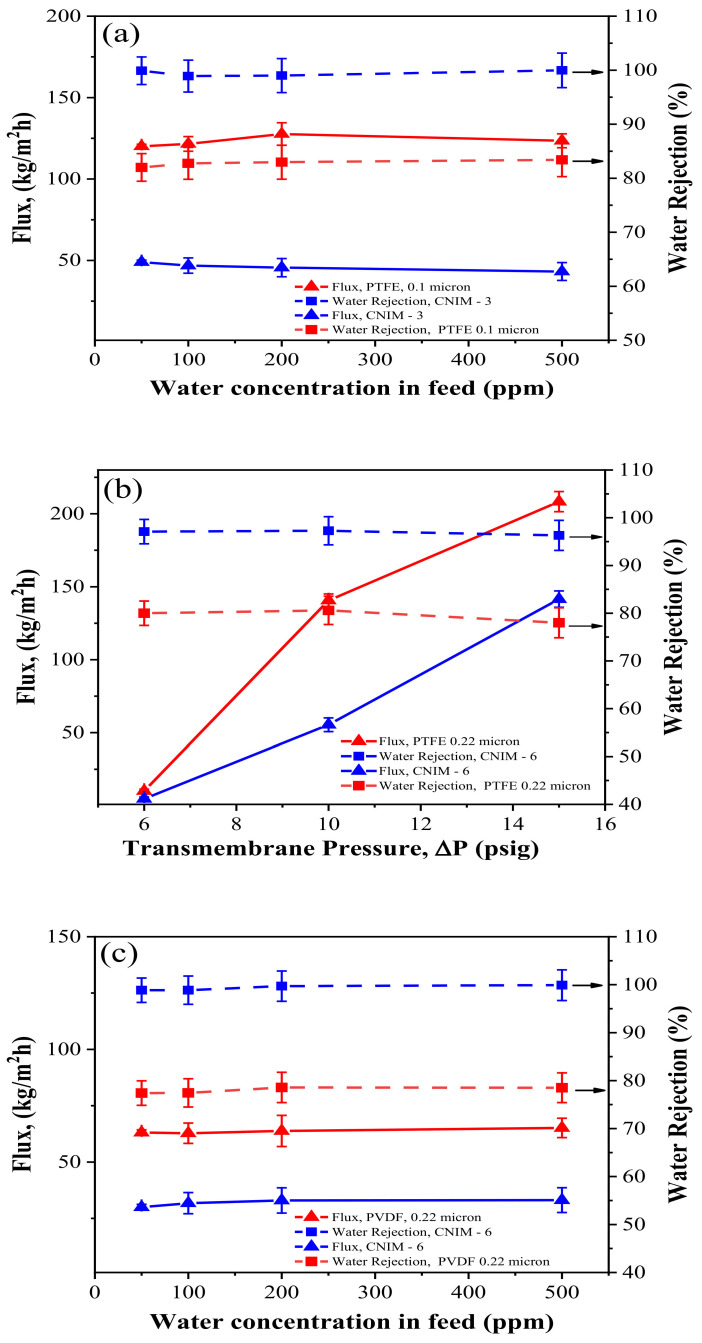
Effect of water concentration on kerosene flux and water rejection at a transmembrane pressure of 10 psig and feed flowrate of 40 mL/min for (**a**) PTFE (0.1 μm) and CNIM-3, (**b**) PTFE (0.22 μm) and CNIM-6 and (**c**) PVDF (0.22 μm) and CNIM-6.

Figure 5 demonstrates the solvent flux and water rejection of various membranes for different water concentrations and at a transmembrane pressure of 10 psig and feed flowrate of 40 mL/min. The water rejection was much higher for CNIMs compared to unmodified PTFE and PVDF membranes. The incorporation of CNTs on the membrane surface did not alter kerosene flux at different concentrations [13]. A water rejection of 97–100% was observed for all optimised CNIMs. For an immiscible water kerosene system, at the trace levels studied here, the concentration variation did not lead to significant changes in the water rejection. This is clearly seen in Figure 5a–c.

## 4. Proposed Mechanism

**Figure 6:**
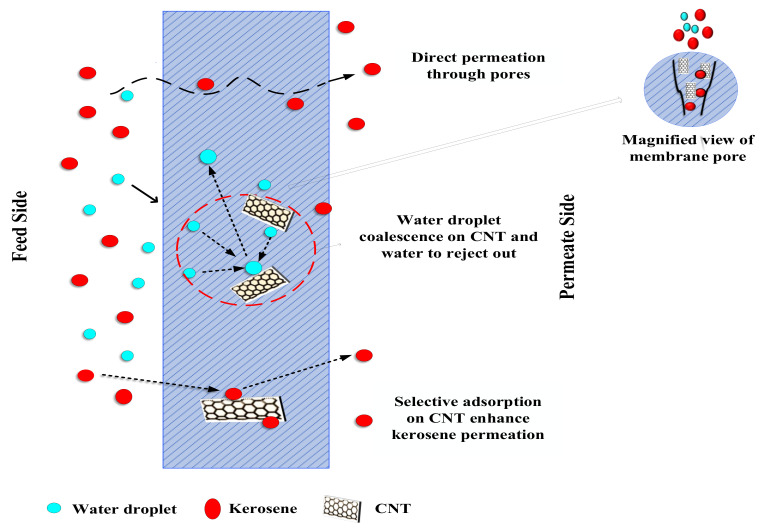
Dewatering mechanism by CNIM.

The mechanism of water removal from kerosene during membrane filtration by hydrophobic CNIM is shown in Figure 6. In addition to improved hydrophobicity, CNTs also provide a significant active surface for partitioning the kerosene phase [14,15,23,29]. Rapid adsorption-desorption on the CNT surface resulted in active diffusion of the kerosene, which increased permeate flux [16,23,24,30,31,32,33,34]. On the other hand, micro/nano water droplets quickly rolled and coalesced due to repulsive forces on CNTs, during which they interacted with one another and began to assemble into larger droplets [4,5,7,8,9,10]. This enhanced water separation during the filtration process [11,12,21,25,35,36,37,38].

## 5. Conclusions

The CNIM-based membrane filtration demonstrated high performance in removing the trace amount of contaminated water. The CNIM has demonstrated ~99% water separation efficiency for the kerosene–water system and significantly improved flux at much lower pressure. The CNTs’ oleophilic and hydrophobic properties allowed kerosene to permeate through the membrane by wetting the sidewalls of the CNTs at various transmembrane pressures. Furthermore, the micro/nanoscale rough features of the CNIM surfaces had a significant impact on the kerosene–water separation. The total flux dropped as the concentration of CNTs increased, yet pure kerosene flux was measured on the permeate side. Surface roughness and water contact angles increase as CNT loading increases, enhancing membrane hydrophobicity. In this work, the partial blocking of pores and the filtering mechanism employing CNIM are thoroughly investigated. This unique development is anticipated to be used for numerous applications, including fuel purification, wastewater treatment, oil spill clean-up, and separation of commercial emulsions.

## Figures and Tables

**Table 1:** Water contact angle for prepared membranes and the results of water rejection.

Membrane	Contact Angle	% Water Rejection *
0.1-micron PTFE	122 ± 2°	83.40
0.22-micron PTFE	112 ± 2°	80.60
0.22-micron PVDF	92 ± 2°	79.00
CNIM-3 (0.1-micron PTFE)	133 ± 2°	99.97
CNIM-6 (0.22-micron PTFE)	130 ± 2°	97.27
CNIM-6 (0.22-micron PVDF)	130 ± 2°	99.9

* Kerosene water system: Feed water concentration 500 ppm, Transmembrane pressure (TMP) 10 psig, Flowrate 40 mL/min.

**Table 2:** Effect of CNTs concentration on membrane performances.

Membrane Performance	PTFE 0.1 Micron			PTFE 0.22 Micron			PVDF 0.22 Micron		
	CNIM-2	CNIM-3	CNIM-6	CNIM-3	CNIM-6	CNIM-8	CNIM-3	CNIM-6	CNIM-8
Water Contact Angle (°)	130 ± 2	133 ± 2	134 ± 2	125 ± 2	130 ± 2	132 ± 2	123 ± 2	130 ± 2	131 ± 2
Flux (kg/m^2^ h)	56.18	43.221	36.83	62.76	55.44	43.12	37.65	33.12	21.12
Water rejection (%)	95.34	99.97	100	94.10	97.27	98.00	93.00	99.90	99.97

## Data Availability

Not applicable.
